# Color improves edge classification in human vision

**DOI:** 10.1371/journal.pcbi.1007398

**Published:** 2019-10-18

**Authors:** Camille Breuil, Ben J. Jennings, Simon Barthelmé, Nathalie Guyader, Frederick A. A. Kingdom

**Affiliations:** 1 McGill Vision Research, Department of Ophthalmology, Montréal General Hospital, Montréal, Québec, Canada; 2 Centre for Cognitive Neuroscience, Division of Psychology, Department of Life Sciences, Brunel University London, London, United Kingdom; 3 GIPSA-lab, Université Grenoble Alpes, CNRS, Grenoble INP, Grenoble, France; Technische Universitat Chemnitz, GERMANY

## Abstract

Despite the complexity of the visual world, humans rarely confuse variations in illumination, for example shadows, from variations in material properties, such as paint or stain. This ability to distinguish illumination from material edges is crucial for determining the spatial layout of objects and surfaces in natural scenes. In this study, we explore the role that color (chromatic) cues play in edge classification. We conducted a psychophysical experiment that required subjects to classify edges into illumination and material, in patches taken from images of natural scenes that either contained or did not contain color information. The edge images were of various sizes and were pre-classified into illumination and material, based on inspection of the edge in the context of the whole image from which the edge was extracted. Edge classification performance was found to be superior for the color compared to grayscale images, in keeping with color acting as a cue for edge classification. We defined machine observers sensitive to simple image properties and found that they too classified the edges better with color information, although they failed to capture the effect of image size observed in the psychophysical experiment. Our findings are consistent with previous work suggesting that color information facilitates the identification of material properties, transparency, shadows and the perception of shape-from-shading.

## Author summary

Our visual environment contains both luminance and color (chromatic) information. Understanding the role that each plays in our visual perception of natural scenes is a continuing topic of investigation. In this study, we explore the role that color cues play in a specific task: edge classification. We conducted a psychophysical experiment that required subjects to classify edges as « shadow » or « other », depending on whether or not the images contained color information. We found edge classification performance to be superior for the color compared to grayscale images. We also defined machine observers sensitive to simple image properties and found that they too classified the edges better with color information. Our results show that color acts as a cue for edge classification in images of natural scenes.

## Introduction

Edges are pervasive features of natural scenes. They can result from a number of causes: object occlusions, reflectance changes, texture changes, shading, cast shadows and highlights, to mention the main varieties. The first three of these constitute changes in material properties, while the last three, namely shading, cast shadows and highlights, constitute changes in the intensity of illumination.

Gilchrist and colleagues were one of the first research groups to point out the importance of classifying edges into material and illumination, in their case to estimate the lightnesses (perceived reflectances) of surfaces [1,2]. There are multiple cues to help distinguish material from illumination [3], one of which, color, would on *a priori* grounds be expected to be useful. In the natural visual world color variations tend to be material in origin, whereas luminance variations tend to be either material or illumination, thereby privileging color over luminance as a potential cue for edge classification [4]. As a result the “color-is-material” assumption has been exploited by computer algorithms tasked with segmenting images of natural scenes into their material and illumination layers [5–7]. Using artificial laboratory stimuli, color information has been shown to facilitate shadow identification [8] and shape-from-shading [9], in ways that are in keeping with the color-is-material assumption. However with natural scenes, while there is evidence that human vision benefits from color in *identifying* edges [10], there is to date no psychophysical evidence that humans similarly benefit from color when *classifying* edges.

We hypothesized that if color acts as a cue for edge classification in natural scenes, observer performance should be better for color compared to grayscale images. Another prediction is that the superiority of color over grayscale will decrease with stimulus size, since larger stimuli contain more contextual cues to help the task thus marginalizing any benefit of color. To test our predictions, we measured the ability of human observers to categorize edges into shadow or material in both color and grayscale images, using three sizes of image patch. To evaluate if simple features related to color are sufficient to account for human performance in the task, we defined a variety of machine observers in the form of Fisher linear classifiers sensitive to simple image properties, and measured their performance when classifying the same edge images.

## Results

### Psychophysical experiment

10 participants (2 females, age 20–40), having normal or corrected visual acuity and normal color vision, took part in the edge categorization task. They were asked to classify briefly presented edges located at the center of each image as “shadow” or “other”. Hence, this was a single-interval forced-choice task. We compared their performance for color and grayscale versions of three different sizes of edge images extracted from the images of natural scenes.

#### Stimuli

**Fig 1:**
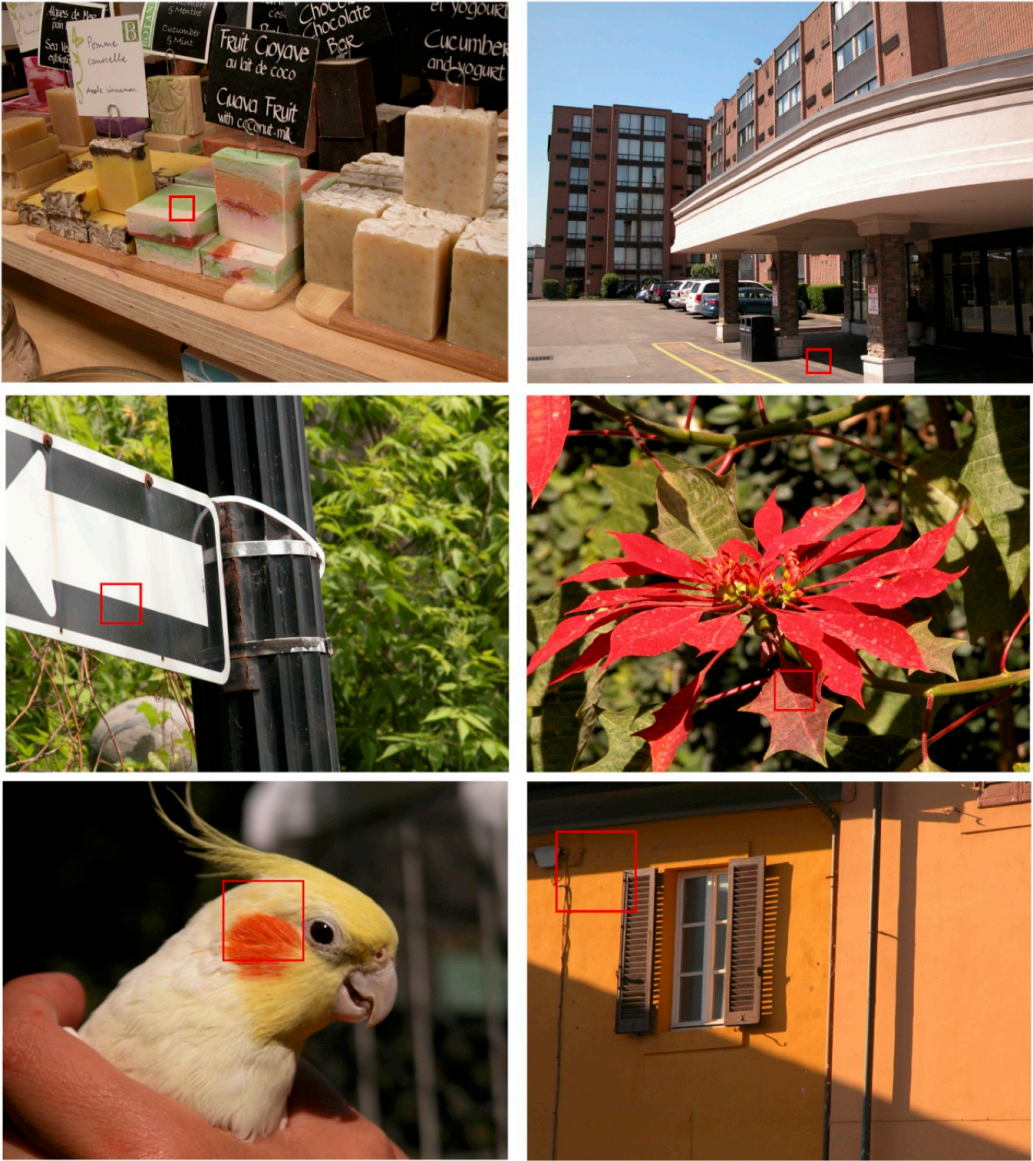
Sample edge stimuli in their original context. Left column: edges from the material category. Right column: edges from the shadow category. Rows top-to-bottom: sample edges of sizes small, medium and large.

**Fig 2:**
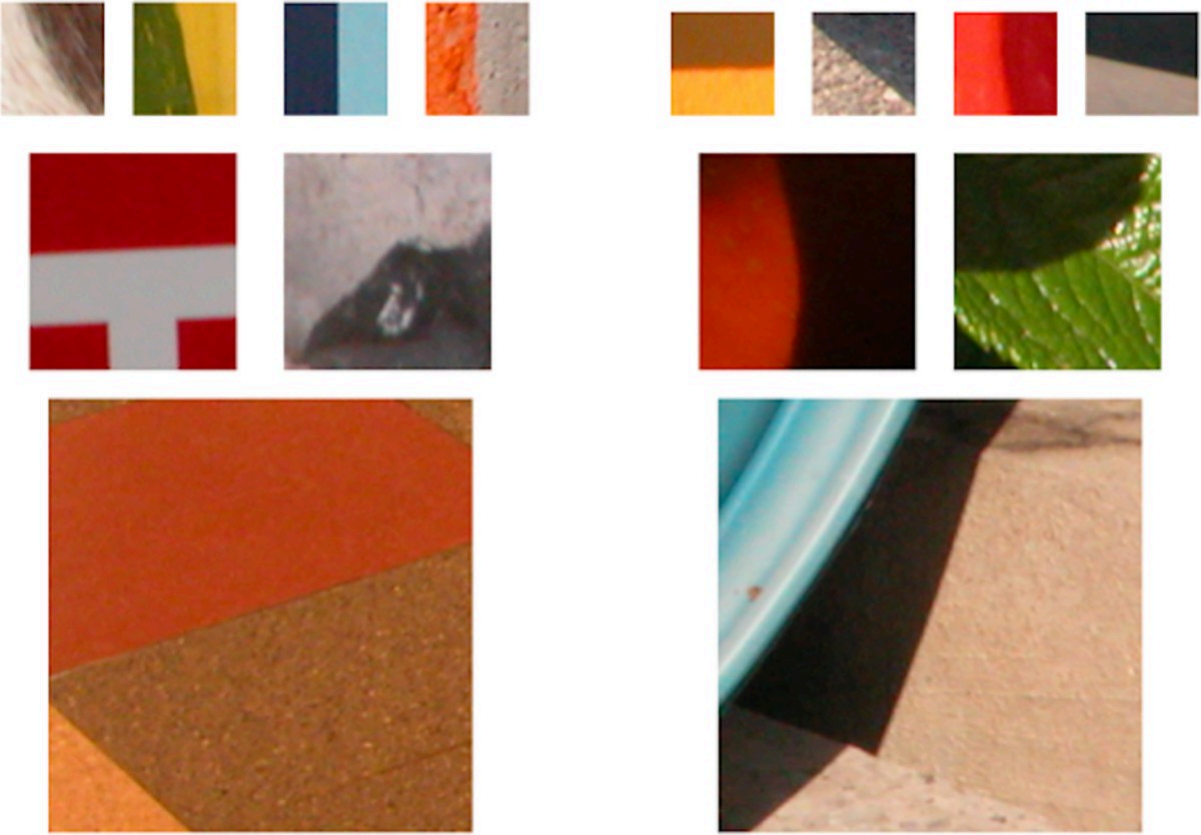
Sample edge stimuli. Left: edges from the material category. Right: edges from the shadow category. Rows top-to-bottom: sample edges of sizes small, medium and large.

Stimuli were images from the publicly available McGill Calibrated Color Image database [11]. Edges that were either ‘pure’ shadows or ‘pure’ material were selected by visual inspection of the whole image. Pure shadow edges were defined as changes *only* in illumination, while pure material edges were defined as changes *without* change in illumination. Square images of different sizes (small: 72 x 72, medium: 144 x 144 and large: 288 x 288 pixels, corresponding approximately to 2.5 x 2.5, 5 x 5 and 10 x 10 degrees of visual angle in the viewing conditions of the experiment) each centered on an edge were extracted using a custom software tool with a cursor. Representative patches of images from our database are illustrated in Figs 1 and 2. The outside-edge of each stimulus was smoothed by applying a circular mask of the same diameter as the stimulus convolved by a Gaussian filter of size 12 x 12 pixels and standard deviation 30 pixels.

#### Color space

To determine if color improves observer classification performance, we compared images containing both luminance and color information with images containing only luminance information. For simplicity we will refer to the former as “color” images and the latter as “luminance-only” images. For the color images we used the untransformed camera images. To create the luminance-only images the following procedure was employed. After a gamma-correction of the monitor’s RGB outputs, the RGB values of the original camera images were first transformed into L (long-wavelength-sensitive), M (medium-wavelength-sensitive) and S (short-wavelength-sensitive) retinal cone-receptor responses, using the measured spectral emission values of the monitor RGB phosphors and the LMS cone sensitivities of human vision established by Smith & Pokorny (1975) [12]. LMS responses were then transformed in a three-dimensional color-opponent space constituted of a luminance axis (Lum), which sums the outputs of the L and M cones, and two chromatic axes (L/M and S/(L+M)), along which the relative excitations of the three cone types vary while the luminance remains constant. Formally, the following transformations of the LMS cone excitations were used [13–15]:

Lum=L+M (1)

$$L/M=\frac{L-\alpha M}{L+M}$$ (2)

$$S/(L+M)=\frac{2S-\beta (L+M)}{2S+\beta (L+M)}$$ (3)

where *α* and *β* are monitor-specific parameters (*α* = 1.33, *β* = 0.14).

**Fig 3:**
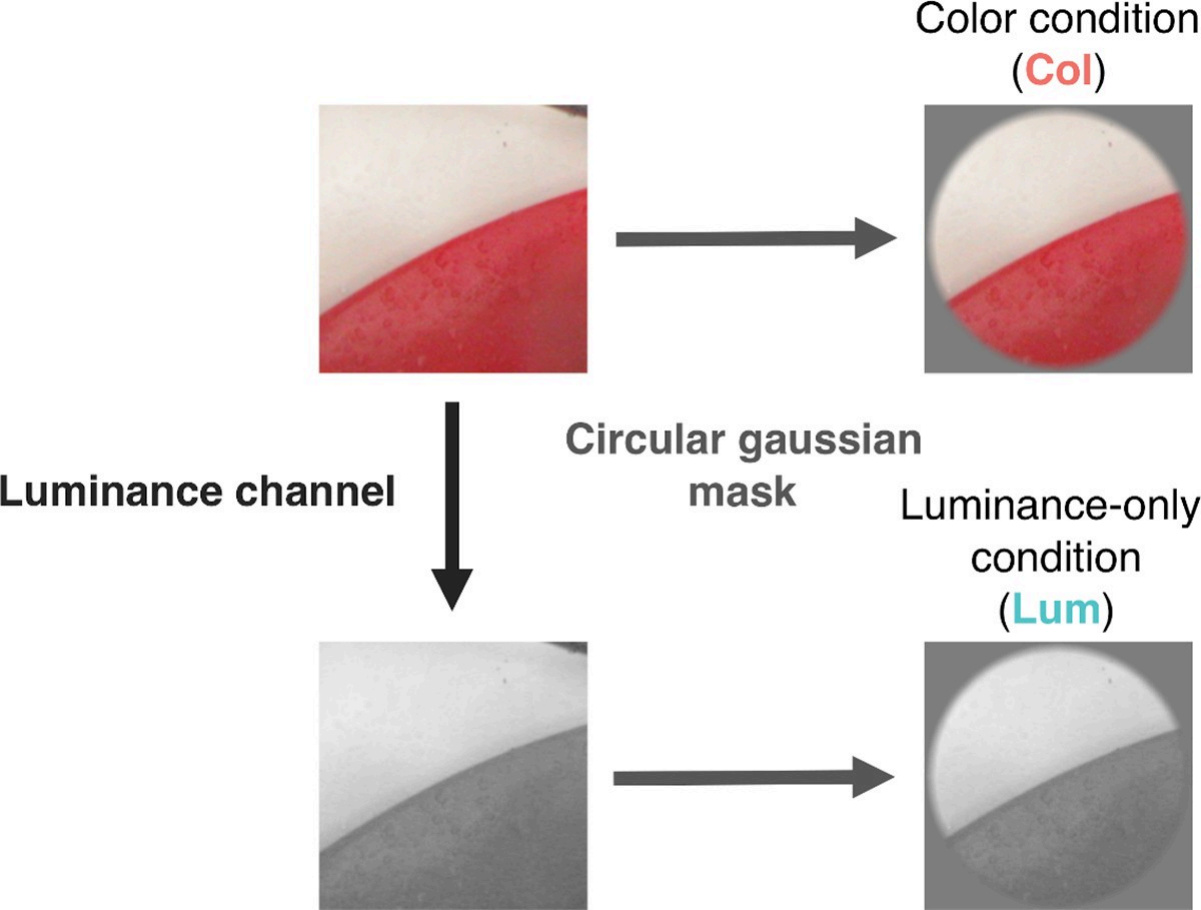
Schematic view of the conversion of the original color image to a luminance-only image and the conversion of both types of image to circular images with a gaussian edge blur. Top right, color condition (Col); bottom right, luminance condition (Lum).

The projection of a pixel in this color space onto the luminance axis preserves the luminance properties of the pixel while removing its chromatic content. Note that this method removes the color content of the image rather than converts it into luminance. A schematic view of the processing chain of the original color image to obtain a color or a luminance-only stimulus is given in Fig 3.

#### Procedure

Observers were seated 57 cm in front of the monitor screen. Head position was stabilized by the use of a chin rest. On each trial a stimulus was briefly presented for 500 ms in the center of the screen. We employed a single-interval forced-choice task in which observers were asked to classify each edge as “shadow” or “other”, by pressing a key on a computer keyboard. The label “other” was deliberately chosen to minimise the semantic difficulty participants might experience in selecting the non-shadow category from the range of possible material edges. For example, we did not use the label “material” because participants might not consider objects to be materials and we did not use “object” because texture edges, paint and stain might not be considered to be objects.

The experiment was divided into 12 blocks of 50 trials, each block containing stimuli of just one of the three sizes, and either color or luminance-only. For each size and each edge category, the observer was presented with 50 color and 50 luminance-only stimuli. The stimuli from our database were randomly assigned as color or luminance-only for each participant, in other words no stimulus was used for both color and luminance-only conditions. Following a training session of 8 trials with feedback, no feedback was given to participants during the test sessions.

#### Data analysis

Single-interval forced-choice tasks tend to be susceptible to response bias, for example in our task there might be an overall tendency for the observer to respond “other”. To take into account the effects of any response bias we converted the data into the signal-detection-theory measure of sensitivity d’ (“d-prime”) [16]. Responses of participants were converted into proportions of Hits (pH) and False Alarms (pFA), where a Hit was defined as an “other” response when a material edge was present and a False Alarm an “other” response when a shadow was present. d’ was then calculated by converting pH and pF into z scores and then taking the difference, thus:

$$d^{\prime }=z(pH)-z(pFA)$$ (4)

Measures of the observers’ response bias towards responding to “other” Or “shadow” are given in S1 Fig.

#### Color improves performance

**Fig 4:**
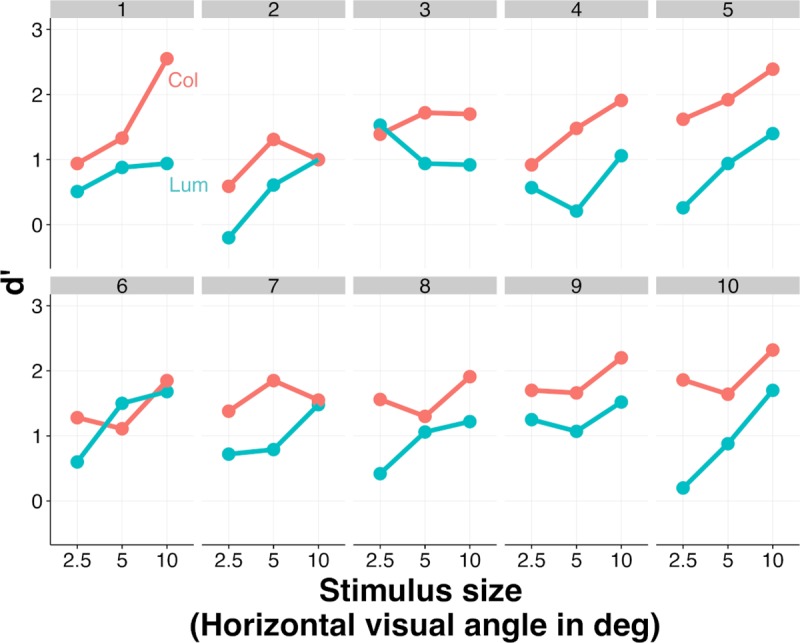
Performance for each of the 10 observers in the classification task, for both color (Col) and luminance-only (Lum) conditions. Results are expressed in terms of d’ values and are show for the different sizes of stimuli.

**Fig 5:**
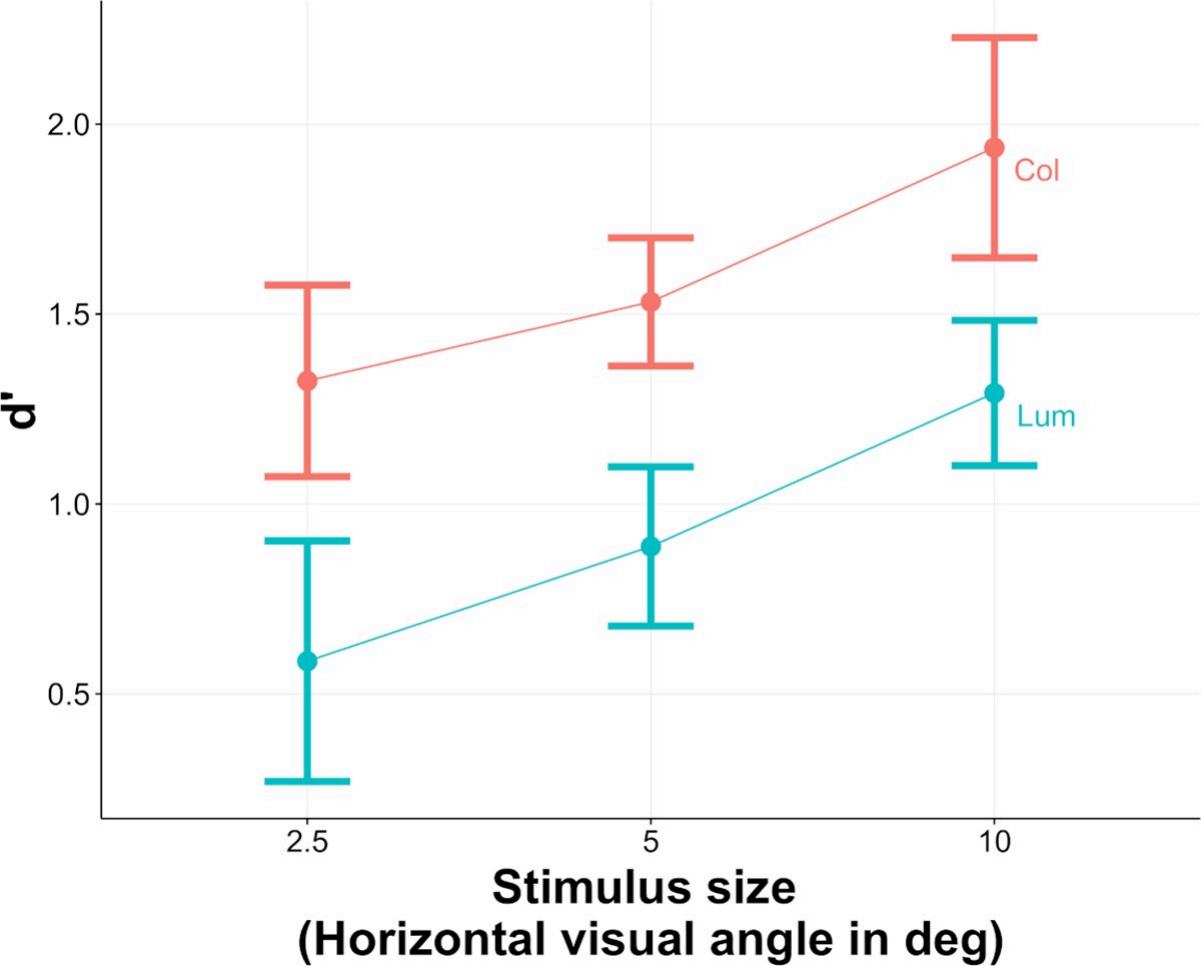
Mean across-observer performance in the classification task for the color (Col) and luminance-only (Lum) conditions, for the three size of stimuli. Results are expressed in terms of d’ values. Error bars are standard errors of the mean d’ values across observers.

Fig 4 shows the d’ values for individual observers and Fig 5 the across-observer average performance (mean d’) for both the color (Col) and the luminance-only (Lum) conditions (red and blue curves), as a function of image size. As can be seen edge classification performance improves with image size and is superior for the color compared to luminance-only condition.

Fig 5 illustrates the effect of both stimulus size and color vs. luminance-only information averaged across observers. As one can see performance significantly improves with size and is superior when color information is present. This suggests that color is an informative perceptual cue for helping disambiguate shadows from material changes. The difference in performance between the color and luminance-only conditions appears to be more-or-less constant, i.e. independent of image size.

### Machine observers

#### Classifiers

We consider here a model binary classification task in which the edge category can assume one of two values, which we represent with the nominal variable e ∈ {shadow,other}. To make a decision regarding the value of e, the classifier takes into account certain image properties, s. We assume a classifier [17,18] that performs the task optimally using the available information. Assuming the stimulus is a shadow edge, the probability of answering correctly is given by P(μ>0), where $\mu =\frac{P(s|e=shadow)}{P(s|e=other)}$ is a decision variable.

We assume that the decision is based on a linear combination of image properties and we use a Fisher’s linear discriminant analysis to test whether including color properties in the model predicts better classification performance.

#### Image properties

**Image pre-processing and region labeling.** We used the same images as those employed in the psychophysical experiment. For each image we hand-marked the position of the edge and then rotated the image so that the edge was approximately centered and horizontally oriented. Each image was then partitioned into 3 regions: L_1_, L_2_ and R, where R was defined as the 10 central pixel rows, L_1_ the region above R, and L_2_ the region below R.

**Luminance measurements.** We first defined the luminance of each pixel as L+M and then normalized the pixel values, such that each image spanned the range from 0 to 1. We then calculated three measures taken from the computational vision literature and employed in several previous studies on surface segmentation [19–21]. The first is based on Michelson contrast, calculated as follows:

$$C_{Lum}=\frac{\left|{Lum}_{1}-{Lum}_{2}\right|}{{Lum}_{1}+{Lum}_{2}}$$ (5)

where Lum_1_ and Lum_2_ are the mean values of luminance of pixels of regions L1 and L2 respectively. Each patch was oriented so that Lum_1_>Lum_2_ (this assignment rule is arbitrary and has no effect on the results). The second measure is based on mean luminance:

$$m_{Lum}=\frac{{Lum}_{1}+{Lum}_{2}}{2}$$ (6)

**Fig 6:**
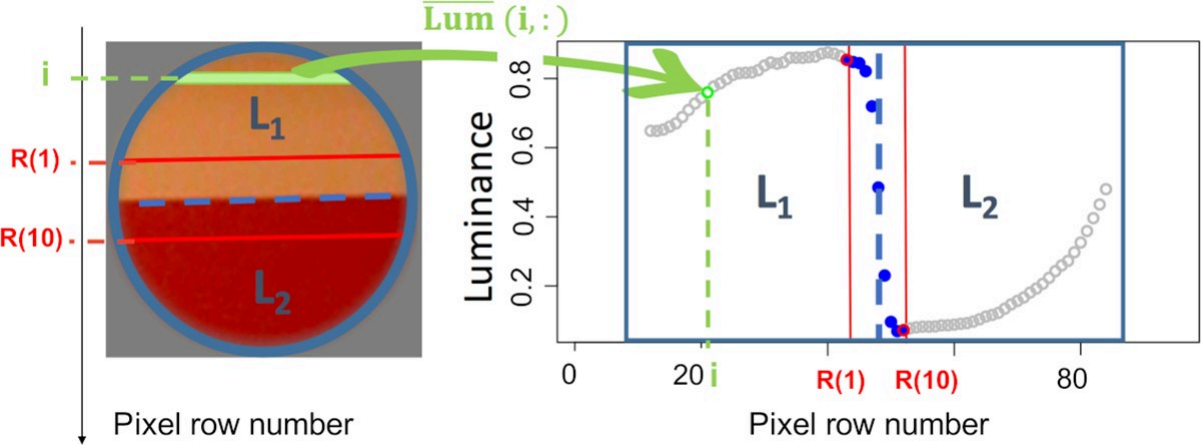
A: An edge image partitioned. For each pixel of the image localized at the row i and the column j we can compute a value of luminance, Lum(i,j). B: One-dimensional profile of the edge image obtained by computing the mean normalized luminance value of each patch row i, $\bar{Lum}(i,:)$.

The third is based on contrast difference:

$$\sigma_{Lum}=|\sigma (L_{1})-\sigma (L_{2})|$$ (7)

where σ(L_1_) and σ(L_2_) are the standard deviation of values of luminance of pixels of regions L_1_ and L_2_. Finally, to quantify the blur of the edge, we computed a fourth measure corresponding to the slope of the luminance transition at the edge. We followed the method proposed by Vilankar et al. (2014) [22] to convert the two-dimensional edge patches to one-dimensional edge profiles and then extracted a measure of slope. The slope (ρ_Lum_) was computed as the mean change from the mean value of luminance of two extreme rows of pixels of the region R:

$$\rho_{Lum}=\frac{\bar{Lum}(R(10),:)-\bar{Lum}(R(1),:)}{9}$$ (8)

where R(1) is the first row of the region R and R(10) the last row of region R. An illustration of the method of image partition and the corresponding luminance edge profile is given in Fig 6.

The average one-dimensional profiles of edges from each size and category are given in S2 Fig. The average slope of the material edges was slightly different from the average slope of the shadow edges.

**Color measurements.** For this analysis we went further than that used in the psychophysics experiment where only edges with- and without color information were compared. Here we wanted to compare the contribution to edge classification of the modelled three post-receptoral color-opponent mechanisms of the human visual system. These mechanisms comprise a luminance mechanism and two color mechanisms, one that compares the activity in the L and M cones, often termed the ‘red-green’ mechanism, the other that compares the activity of the S with the sum of the L and M cones, often termed the ‘blue-yellow’ mechanism. To model the responses of these mechanisms to natural-scene image information, we used the pixel-based definitions of color-opponent responses given in the previous section (Eqs 2 and 3). As pointed out by Olmos and Kingdom (2004) [7], these definitions are arguably superior to those based on cone contrasts such as the DKL color space [23] when applied to natural scene stimuli. The reason is that the normalization operates on a pixel-by-pixel, i.e. local basis rather than via the image as a whole, in keeping with the idea that cone adaptation is a spatially local rather than global process [7].

As in DiMattina et al. (2012) [21], we employed two measures of the difference in color content across the edge. First, the ‘red-green’ difference, Δ_L/M_:

$$\Delta_{L/M}=|{L/M}_{1}-{L/M}_{2}|$$ (9)

where L/M_1_ and L/M_2_ are the mean L/M pixel opponency values in regions L_1_ and L_2_ respectively. Second, and correspondingly, the ‘blue-yellow’ difference, Δ_S/(L+M)_:

$$\Delta_{S/(L+M)}=|{S/(L+M)}_{1}-{S/(L+M)}_{2}|$$ (10)

To quantify the potential usefulness of the above luminance and color information in the categorization task, we determined the performance of various classifiers. Each classifier was defined by its use of a specific combination of image properties (with or without color). We especially wanted to test if the addition of color information improved classifier performance.

#### Fisher linear discriminant analysis (LDA)

We used a Fisher Linear Discriminant Analysis (LDA) [24] to find a linear combination of image properties that best separates our two edge classes across both image size and color content. LDA is based on linear transformations that maximize a ratio of “between-class variance” to “within-class variance” with the goal of reducing data variation in the same class and increasing the separation between classes.

Consider a set of n images and observations s = {s_1_,…, s_k_} for each image. The classification problem is to find a good predictor for the class e of any sample of the same distribution (not necessarily from the training set) given only the n observations s. To use this approach, we assume that the conditional probability density functions P(s|e = shadow) and P(s|e = other) are both normally distributed with mean and covariance parameters (μ_0_,Σ_0_) and (μ_1_,Σ_1_). Then, the Bayes optimal solution is to predict edges that are shadows if the decision criterion μ>1, or equivalently if log(μ)>0 where

$$\begin{array}{c} \log\left(\mu \right)=\log\left(\frac{P(s|e=shadow)}{P(s|e=other)}\right)=\log\left(\frac{\det{\left(\Sigma_{1}\right)}^{1/2}}{\det{\left(\Sigma_{0}\right)}^{1/2}}\right)\\ =\frac{1}{2}[{\left(s-\mu_{1}\right)}^{T}\Sigma_{1}^{-1}(s-\mu_{1})-{\left(s-\mu_{0}\right)}^{T}\Sigma_{0}^{-1}(s-\mu_{0})] \end{array}$$ (11)

An additional assumption required to use LDA is that the class covariances are equal (Σ_0_ = Σ_1_ = Σ). As a consequence, several terms cancel:

$$s\Sigma_{1}^{-1}s=s\Sigma_{0}^{-1}s$$ (12)

and $s\Sigma_{i}^{-1}\mu_{i}=\mu_{i}\Sigma_{i}^{-1}s$ because Σ_i_ is symmetric. The decision criterion log(μ) simplifies as

$$\log\left(\mu \right)={s\Sigma }^{-1}(\mu_{1}-\mu_{0})-\frac{1}{2}(\mu_{0}\Sigma^{-1}\mu_{0}-\mu_{1}\Sigma^{-1}\mu_{1})$$ (13)

If we denote w = ∑^−1^(μ_1_−μ_0_) and $c=\frac{1}{2}(\mu_{0}\Sigma^{-1}\mu_{0}-\mu_{1}\Sigma^{-1}\mu_{1})$, a simpler expression of decision criterion becomes w.s>c, where w.s is simply the dot product of vector w and observations s. This means that the criterion of an input s being in a class e is purely a function of this linear combination of the known observations.

If we find that humans outperform our linear classifiers, we can conclude that humans are either using information that we have not considered or that they combine the information differently. Such an outcome would suggest that classifier performance could be improved by including more stimulus information, or by using a possibly non-linear classifier.

#### Classifier performance

We evaluated a large set of classifiers, each of them defined by a subset of the four luminance images properties (C_Lum_, m_Lum_, σ_Lum_ and ρ_Lum_) with or without adding color properties (Δ_L/M_ and Δ_S/(L+M)_). The performance of classifiers were measured for both the entire images set and for each size independently. A summary of all the results is given in S3 Fig.

We estimated classifier performance using a confusion matrix and computed d’ values to compare classifier to human observer performance.

**Fig 7:**
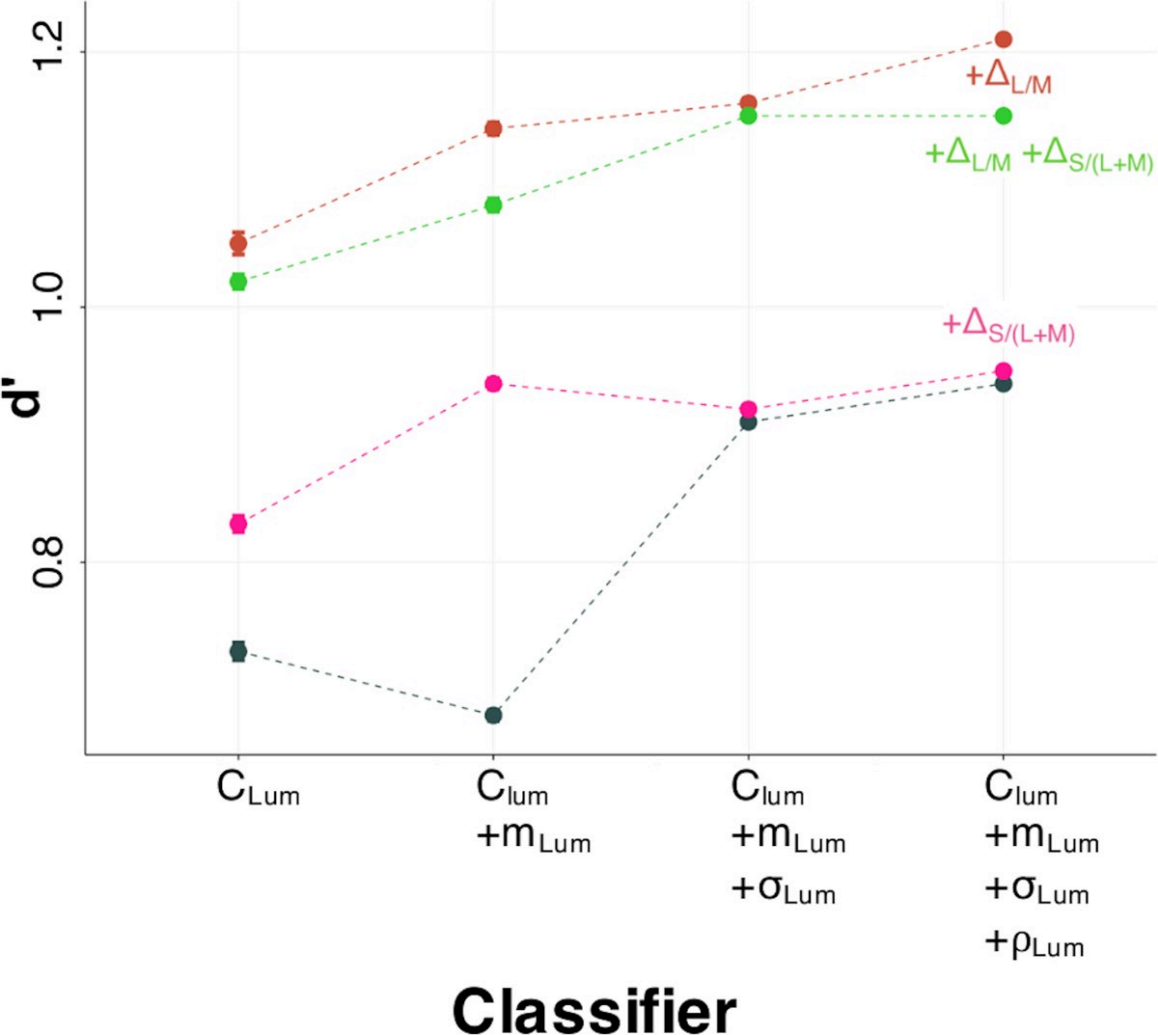
Classifier performance as a function of number of image properties. Image properties were first ranked by their individual d’ measures and then adding in order from highest to lowest. The reference classifier (in grey) only integrates luminance properties, while +Δ_L/M_ adds the Δ_L/M_ measures, +Δ_S/(L+M)_ adds the Δ_S/(L+M)_ measures and +Δ_L/M_+Δ_S/(L+M)_ adds both color measures. Intervals correspond to lower and upper 95^th^ percentile confidence interval based on parametric bootstrap simulations (n = 1000).

As illustrated in Fig 7, all our classifiers performed better with color information. This shows that there is a sound physical basis for the improved human observer classification performance we observed for the color compared to luminance edges. The results of the classifiers which include both color properties, Δ_L/M_ and Δ_S/(L+M)_, are quite similar to those of the ones including only Δ_L/M_. Moreover, as illustrated on Fig 7, the inclusion of Δ_S/(L+M)_ measure only improves classifier performance when just considering C_Lum_ or C_Lum_+m_Lum_, suggesting that, contrary to the Δ_L/M_ measure, the Δ_S/(L+M)_ measure is not really helpful for the classification.

### Comparison of human and classifier performance

**Fig 8:**
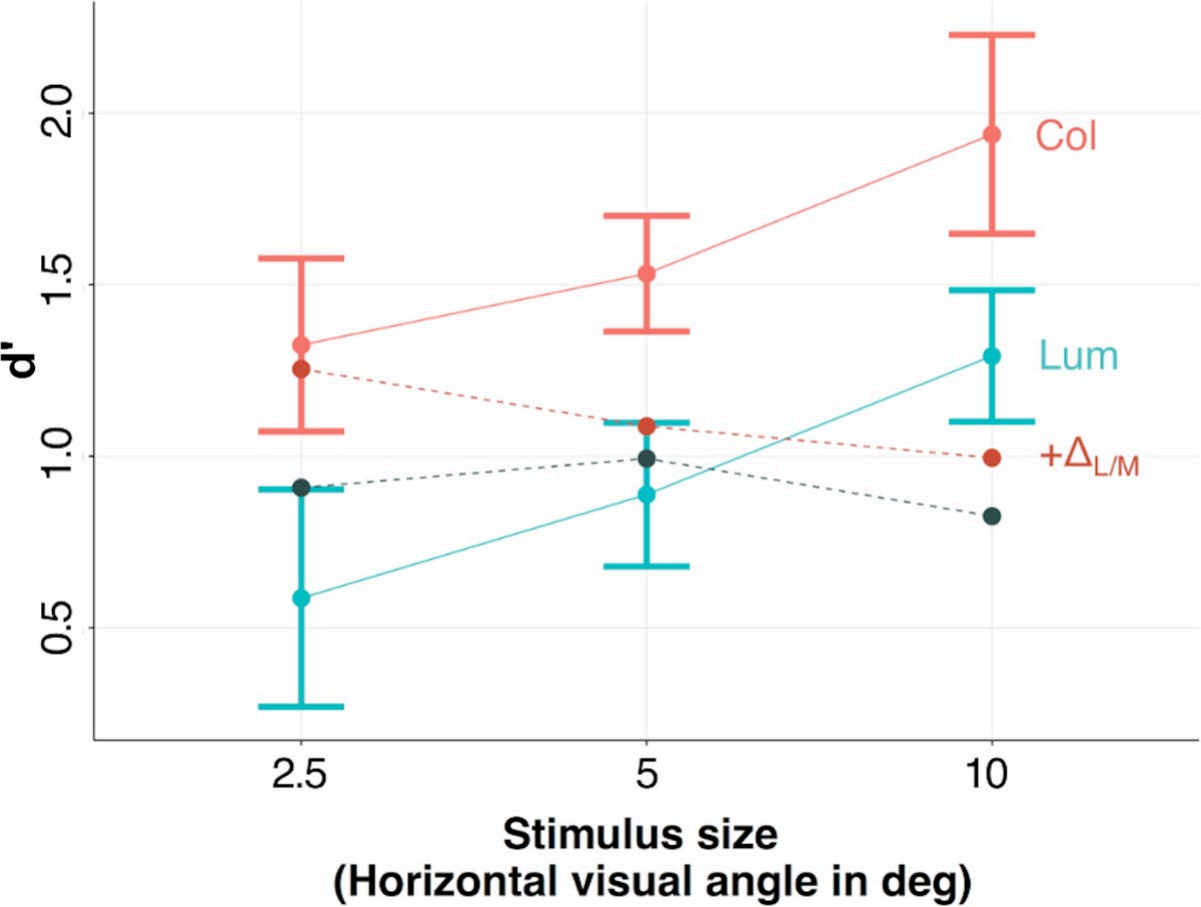
Comparison of human observers and classifiers for color and luminance-only conditions.

Fig 8 compares the performance of the classifiers with that of the human observers. In the case of the luminance-only condition (Lum) the classifier is referenced as C_Lum_+m_Lum_+σ_Lum_+ρ_Lum_ whereas in the color condition (Col) it is C_Lum_+m_Lum_+σ_Lum_+ρ_Lum_+ Δ_L/M_(+ Δ_S/(L+M)_), as it includes the Δ_L/M_ measure (and Δ_S/(L+M)_).

We see that the classifier correctly captures the experimentally observed improvement of performance with color information. However, it fails to account for the effect of stimulus size that we observed with humans, especially for the larger stimuli. This suggests that subjects must be making use of spatial cues not considered here. The image properties we selected for our classifier are relatively simple visual features, yet interestingly, are able to predict the improvement of performance with color.

## Discussion

To our knowledge no psychophysical studies have directly considered the usefulness of color cues for classifying natural-scene edges. In the present study we conducted a psychophysical experiment to evaluate the role of color information in material-vs.-illumination edge categorization in part-images of natural scenes. Our results show a consistent improvement in classification performance when the color information in the images was available. This suggests that the visual system does indeed use color information for edge classification, presumably at a relatively early stage of visual processing. We expected that the difference between color and luminance would decrease as the images became larger because with more context the visual system might rely less on color to perform the task. However, Fig 5 suggests that, for human observers, color is no less useful for large than for small stimuli.

Cue combination for edge classification has been mainly considered in the computer vision literature, but the models that have emerged have not been directly compared with human data in a controlled psychophysical experiment. We therefore tested a classifier sensitive to various basic image properties to see if model performance paralleled that of our human observers. All the tested classifiers worked well and performed significantly better with color information. However, as illustrated in Fig 8, the improvement of classifier performance with added color information was not as large as for human observers and our classifiers failed to capture the effect of image size observed with our human observers. Indeed, while the performance of human observers was improved by size and color, that of classifiers decreases with size, and the difference between color and pure-luminance conditions is less constant. The classifiers we employed used relatively basic properties such as the difference in mean luminance either side of the edge, whereas our human observers were presumably also sensitive to more complex and information-rich color and luminance image properties, such as information about the texture [25–30], shape and spatial orientation of the edge [31–35], as well as higher order contextual information. Indeed, edges rarely occur in isolation, instead in a rich, structured context of other visual information. Although context is an overloaded source of information, it has been shown that contextual information can be extracted quickly [36,37], and is potentially based on low-level feature statistics [38]. Contextual information can enhance object detection and recognition performance [39,40], help disambiguate visual displays [41], and provide prior information on the likely positions of objects and constrain the range of possible objects likely to occur within that context [42]. Natural scenes contain rich contextual information, affecting a variety of recognition-related processes and improving visual performance [43]. In our experiment, contextual information would be most prominent in the larger images and is likely the reason why the performance of the human observers but not classifiers improved with stimulus size.

Moreover, the properties used by classifiers are computed on the whole stimulus, so when the size increases we no longer really capture edge-specific properties as there is more context. Thus we assume that the classifiers performance could be significantly improved by refining the image properties, using more local measures [44–47], which really capture edge-specific properties, and adding other cues useful for edge classification.

Interestingly, our classifiers revealed a different role of Δ_L/M_ and Δ_S/(L+M)_ color information: while the L/M measures were helpful for the task, addition of the S/(L+M) measures did not improve classifier performance. This is consistent with previous studies that the S/(L+M) opponent channel varies with changes in illumination, such as at shadow borders [48], while the L/M system is more robust to illumination changes, thus providing a more reliable cue to material borders [7,13,14,48,49]. Therefore, an interesting direction for future work is to test whether for human observers L/M information is more useful than S/(L+M) information for performing the classification task, as predicted by our classifier model.

In conclusion, for both human and model observers color information facilitates the classification of edges in natural scenes into material versus illumination. For both types of observer, the improvement with color is robust to variations in image size. Our findings highlight the importance of color in the visual analysis of the structural properties of natural scenes.

## Methods

### Ethics statement

The experiment conformed to the Declaration of Helsinki and all participants gave their informed oral consent before participating in the study.

### Apparatus

Visual stimuli were displayed on a 21-inch CRT monitor with a spatial resolution of 1024 x 768 pixels and a refresh rate of 100Hz. The background was set to a neutral gray (RGB = [127,127,127] on a 256 level-scale). Spectro-radiometric calibration was performed on the three phosphors of the monitor using a CS 2000 Konica Minolta spectro-radiometer. Spectra of the three RGB primaries were first measured at their maximum intensity setting and then multiplied with the Judd-revised CIE color matching functions [50] to derive the CIE xyY coordinates of the monitor phosphors which were then used to convert between RGB and color-opponent space. The xyY coordinates of the monitor primaries measured at maximum intensity were 0.6207, 0.3380 and 15.0485 (red); 0.2822, 0.6068 and 57.0515 (green); 0.1495, 0.0683 and 6.9209 (blue).

## Supporting information

S1 Fig Individual response bias. (PDF)

S2 Fig Average one-dimensional edge profiles. (PDF)

S3 Fig Classifier performances. (PDF)

S1 Dataset Psychophysical experiment dataset. (XLSX)
