# Supplementary material for: Color improves edge classification in human vision
Source: PLoS Comput Biol. 2019 Oct 18;15(10):e1007398. doi: 10.1371/journal.pcbi.1007398 (PMC6827913; doi:10.1371/journal.pcbi.1007398)
Supplement: S1 Fig — (PDF) [file pcbi.1007398.s001.pdf]

S1 Figure: Individual response bias

For each participant, we calculated a measure of response bias *c* given by $c = -\frac{z(FA)+z(H)}{2}$. A value of *c* less than 0 indicates that the observer is biased to respond “other” whereas a value greater than 0 indicates that the observer is biased to respond “shadow”. S1Figure shows that most of our participants had a bias towards responding “shadow”, and this bias is larger in the pure-luminance condition than in the color condition.

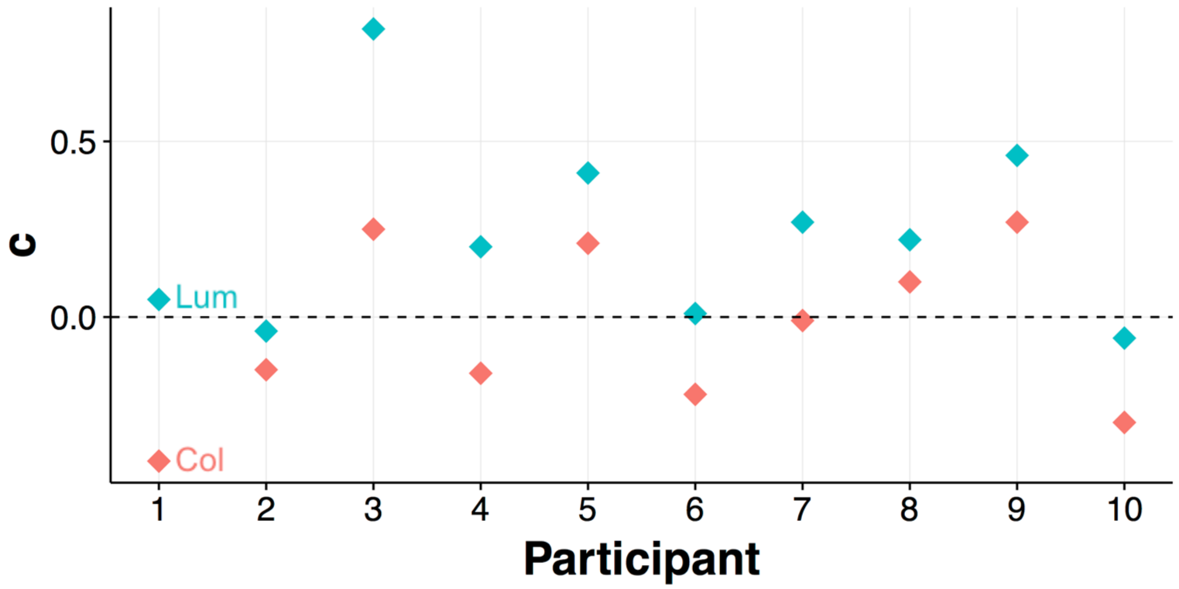

Figure S1. Observer response bias *c* in the psychophysical experiment, for the luminance-only (Lum) and color (Col) conditions.
