# Supplementary material for: Color improves edge classification in human vision
Source: PLoS Comput Biol. 2019 Oct 18;15(10):e1007398. doi: 10.1371/journal.pcbi.1007398 (PMC6827913; doi:10.1371/journal.pcbi.1007398)
Supplement: S2 Fig — (PDF) [file pcbi.1007398.s002.pdf]

S2 Figure: Average one-dimensional edge profiles

We computed the average one-dimensional profile of edges from each size and category. As illustrated on S2 Figure, slope of average material edges was slightly different from the slope of average shadow edges.

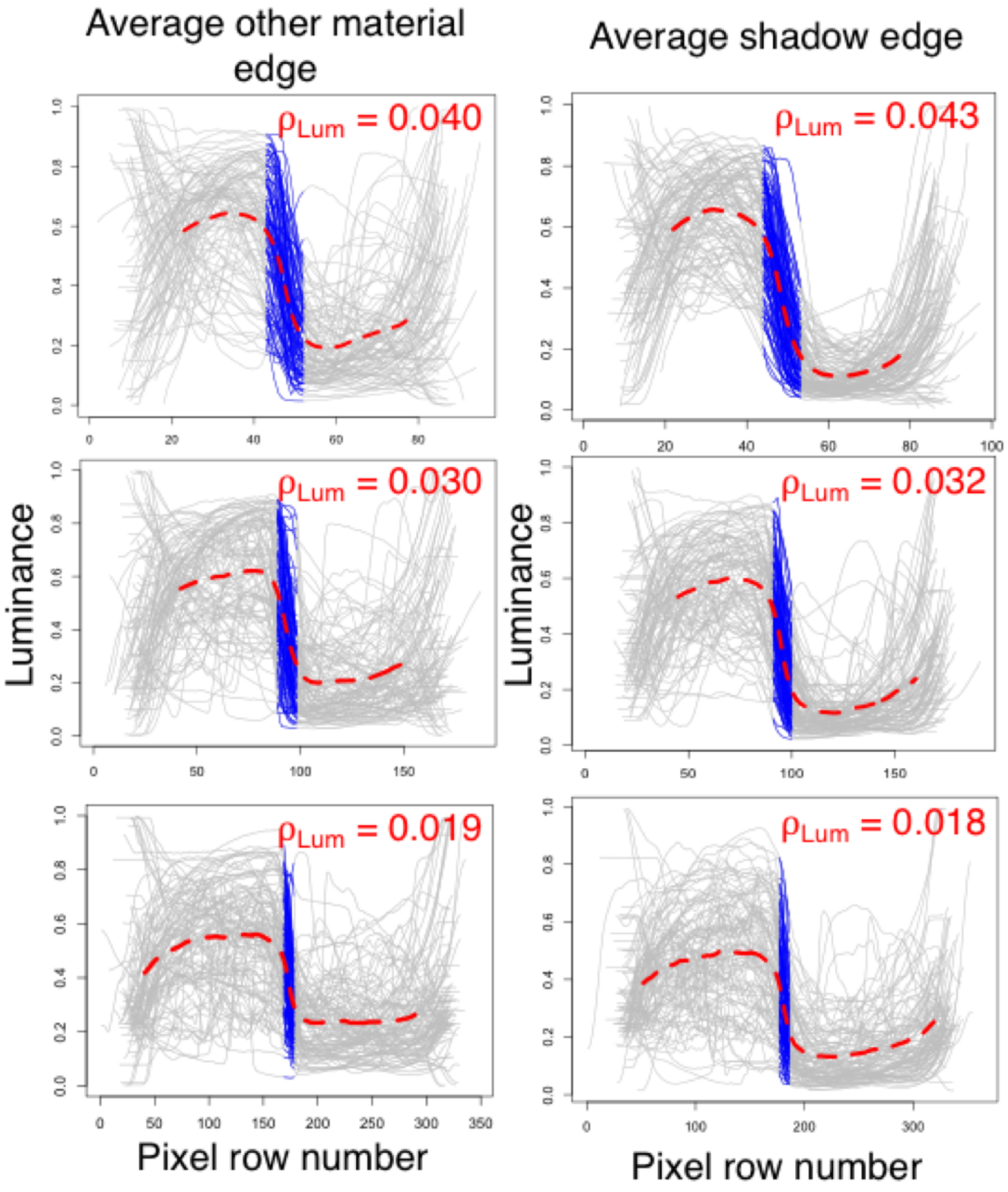

Figure S2 – One dimensional profile of normalized average edge. On the left, edges from the material category. On the right, edges from the shadow category. Each row corresponds to one size of edge patches (small, medium and large). The average edge is in red with all the edges of the subset in gray.
