# Supplementary material for: Color improves edge classification in human vision
Source: PLoS Comput Biol. 2019 Oct 18;15(10):e1007398. doi: 10.1371/journal.pcbi.1007398 (PMC6827913; doi:10.1371/journal.pcbi.1007398)
Supplement: S3 Fig — (PDF) [file pcbi.1007398.s003.pdf]

S3 Figure: Classifier performances

We evaluated performance of our different classifiers for each image size independently. The d’ values we computed are shown in Figure S3. Comparing these results with observers’ performance in our psychophysical experiment, we see that almost all our Bayesian model classifiers fail in reproducing the effect of size observed for our participants.

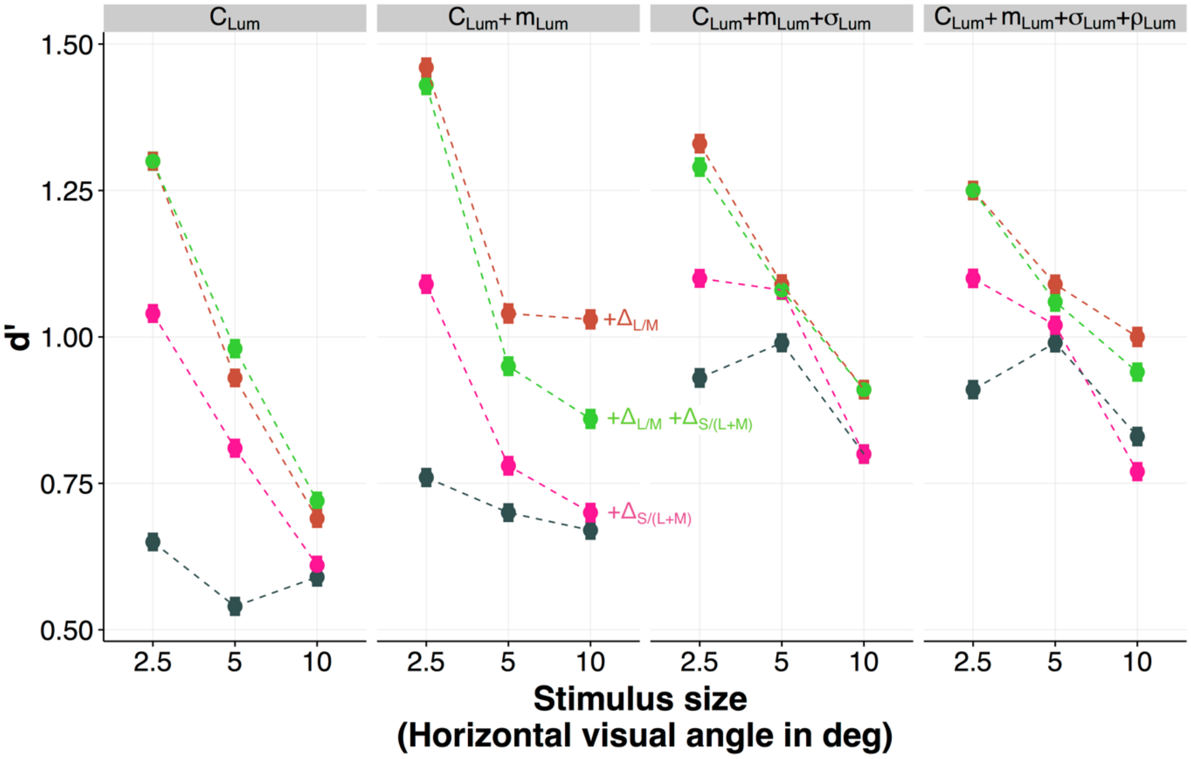

Figure S3 –Summary of classifiers performance expressed in terms of d’ values. Intervals correspond to lower and upper 95th percentile confidence interval based on parametric bootstrap simulations (n=1000).
